# Genetic Diversity Strategy for the Management and Use of Rubber Genetic Resources: More than 1,000 Wild and Cultivated Accessions in a 100-Genotype Core Collection

**DOI:** 10.1371/journal.pone.0134607

**Published:** 2015-07-30

**Authors:** Livia Moura de Souza, Vincent Le Guen, Carlos Bernardo Moreno Cerqueira-Silva, Carla Cristina Silva, Camila Campos Mantello, Andre Ricardo Oliveira Conson, João Paulo Gomes Vianna, Maria Imaculada Zucchi, Erivaldo José Scaloppi Junior, Josefino de Freitas Fialho, Mario Luis Teixeira de Moraes, Paulo de Souza Gonçalves, Anete Pereira de Souza

**Affiliations:** 1 Molecular Biology and Genetic Engineering Center (CBMEG), University of Campinas (UNICAMP), Campinas, SP, Brazil; 2 Centre de Coopération Internationale en Recherche Agronomique pour le Développement (CIRAD) UMR AGAP, Montpellier, Hérault, France; 3 Laboratory of Applied Molecular Genetics, Department of Exact and Natural Sciences, State University of Southwest Bahia (UESB), Itapetinga, BA, Brazil; 4 Agência Paulista de Tecnologia dos Agronegócios (APTA), Pólo Regional Noroeste Paulista, Votuporanga, SP, Brazil; 5 Embrapa Cerrados (EMBRAPA), Planaltina, DF, Brasil; 6 Departamento de Fitotecnia, Faculdade de Engenharia de Ilha Solteira (UNESP)–Universidade Estadual Paulista, Ilha Solteira, SP, Brazil; 7 Rubber Research Advanced Center (CAPSA), Agronomical Institute (IAC), Votuporanga, SP, Brazil; 8 Department of Plant Biology, Biology Institute, University of Campinas (UNICAMP) UNICAMP, Campinas, SP, Brazil; Università Politecnica delle Marche, ITALY

## Abstract

The rubber tree [*Hevea brasiliensis *(Willd. ex Adr. de Juss.) Muell. Arg.] is the only plant species worldwide that is cultivated for the commercial production of natural rubber. This study describes the genetic diversity of the *Hevea* spp. complex that is available in the main *ex situ* collections of South America, including Amazonian populations that have never been previously described. Genetic data were analyzed to determine the genetic structure of the wild populations, quantify the allelic diversity and suggest the composition of a core collection to capture the maximum genetic diversity within a minimal sample size. A total of 1,117 accessions were genotyped with 13 microsatellite markers. We identified a total of 408 alleles, 319 of which were shared between groups and 89 that were private in different groups of accessions. In a population structure and principal component analysis, the level of clustering reflected a primary division into the following two subgroups: cluster 1, which consisted of varieties from the advanced breeding germplasm that originated from the Wickham and Mato Grosso accessions; and cluster 2, which consisted of the wild germplasm from the Acre, Amazonas, Pará and Rondônia populations and *Hevea* spp. The analyses revealed a high frequency of gene flow between the groups, with the genetic differentiation coefficient (G_ST_) estimated to be 0.018. Additionally, no distinct separation among the *H*. *brasiliensis* accessions and the other species from Amazonas was observed. A core collection of 99 accessions was identified that captured the maximum genetic diversity. Rubber tree breeders can effectively utilize this core collection for cultivar improvement. Furthermore, such a core collection could provide resources for forming an association panel to evaluate traits with agronomic and commercial importance. Our study generated a molecular database that should facilitate the management of the *Hevea* germplasm and its use for subsequent genetic and genomic breeding.

## Introduction

Over the last decade, the number of *ex situ* collections of cultivated plants has greatly increased. This increase has occurred partially in response to the major alterations caused by the development of human settlements in the areas of origin of the related wild species. Among the numerous existing collections, those involving perennial crops (either forestry or fruit crops) are especially difficult to establish. Moreover, these collections are expensive to manage due to the large areas that are required to maintain the accessions, such as the *ex situ* collections of grapevines [1], olive trees [2] and apple trees [3]. These difficulties are often exacerbated in perennial crops from tropical or sub-tropical areas due to the velocity with which ecosystems are deteriorating in these regions and the frequent paucity of the financial resources available for preserving biodiversity in the concerned countries. This issue is of particular concern for cultivated trees originating from Asian tropical rainforests (such as *Tectona grandis* [4]), African tropical rainforests (such as *Elaeis guineensis* [5] and *Coffea canephora* [6]) and Amazonian or Atlantic forests (such as *Theobroma cacao* [7] and *Hevea brasiliensis* [8]). *Ex situ* collections have generally been established to save natural populations of cultivated crops from irremediable destruction when the original habitat for the crop has been threatened by deforestation. These collections also aim to provide breeders with genetic resources that are useful for genetic improvement (e.g., related to resistance to biotic or abiotic stress). However, these *ex situ* collections are frequently partially redundant among countries and even among research centers in the same country due to the duplication of accessions and sharing between institutions [9,10]. Such redundancy is even more common for plants exhibiting easy vegetative propagation [11,12]. The genetic characterization of available genetic resources may permit optimization of the use of these resources by grouping a sufficient number of accessions in a core collection to maximize the genetic diversity described in the whole collection.

The rubber tree (*Hevea brasiliensis)* is a quite recent crop, with genetic improvements initiated only a short time ago. Breeders initiated their work on this species in South East Asia at the beginning of the twentieth century after the successful introduction of approximately 20 seedlings into that part of the world. These seedlings originated from more than 70,000 seeds that were collected by Wickham in 1876 near the Tapajos River in the state of Pará, Brazil. These seeds were sent to England and germinated in the Royal Botanical Garden of Kew, London. The few seedlings obtained after rather poor germination (due to the lack of dormancy in rubber seeds) were sent to Malaysia and Ceilon and served as the origin of all of the rubber tree plantations in the region. The multiplication of these plants was primarily performed through seeds and bud grafting from the 1920s onward when this technique was well managed [13]. Almost all of the clonal varieties that are cultivated today, as well as the genotypes that are still under selection, are less than 10 generations removed from their wild ancestors in the Amazonian forest; this time span represents a very small number of generations for a tree that is cultivated in very large areas. Furthermore, the vast majority of these clones originated from the so-called Wickham trees that were introduced into Asia at the end of the nineteenth century. Therefore, the genetic basis of the cultivated material is quite narrow, even if most of the cultivars currently exhibit sufficient allelic diversity to allow for substantial genetic improvement.

The cultivated species *H*. *brasiliensis* originated from the Amazonian forest, where it coexists in various places with some of the ten other species of the genus *Hevea*. All of the *Hevea* species are monoecious and display a preferentially outcrossing habit. Natural interspecific hybridization can occur across the genus and generate gene flow, which has led some authors to consider *Hevea* to be a species complex [14]. Various surveys were performed during the twentieth century with the aim of collecting seeds and budwood from wild rubber trees to increase the genetic pool. One of the first collecting expeditions was conducted under the authority of the Peruvian ministry of agriculture in 1940 in the region of Madre de Dios [15]. In 1974, a joint survey was performed by IRCA (Institut de Recherche sur le Caoutchouc, France) and Embrapa (Empresa Brasileira de Pesquisa Agropecuária, Brazil) in the Brazilian states of Acre and Rondônia [16]. In 1981, the IRRDB (International Rubber Research and Development Board) led an international effort to collect large numbers of rubber tree seeds and budwood from the Brazilian states of Acre, Mato Grosso and Rondônia [17–19]. All of the collected material was dispatched between Brazil, Malaysia and Côte d’Ivoire [20] and then further dispersed in these regions. Later, in an attempt to characterize the diversity with isozymes markers, two natural populations that were close to each other and located near Rio Branco (Acre, Brazil) were sampled and conserved *ex situ* in the Brazilian state of São Paulo [21]. In 1995, a common survey conducted by Embrapa (Brazil) and the RRIM (Rubber Research Institute of Malaysia) was organized to collect seeds from various species of the *Hevea* genus in the Brazilian states of Para and Amazonas [22]. The numerous collected seeds were divided between Malaysia and Brazil. In Brazil, a unique *ex situ* collection containing this material was established in an experimental area from Embrapa near Brasília. To date, the germplasm collected in 1995 has never been described, multiplied or used in breeding programs.

Our objective in the present study was to describe the genetic diversity of the *Hevea* spp. complex available in the main *ex situ* collections of South America. The accessions that were collected during the four surveys described above and conserved in the Brazilian states of Brasília and São Paulo, as well as commercial cultivars and their hybrids were characterized with molecular markers and compared with similar data obtained in a previous study of accessions from an *ex situ* collection in French Guiana. Genetic data were analyzed to describe the genetic structure of the wild populations that were sampled, to quantify the allelic diversity and to suggest the composition of a core collection that would capture the maximum genetic diversity within a minimal sample size.

## Materials and Methods

Data from genotypes that were previously collected and analyzed by Le Guen et al. were included in the present work [23]. These data were added to a set of original data obtained from new samples of vegetal material and analyzed jointly. These new data are described hereafter.

### Plant material

The numbers of accessions per geographical origin and *ex situ* collection site are listed in Table 1. The majority of these accessions were collected during one of the above-mentioned surveys in Peru (region of Madre de Dios) or Brazil (states of Acre, Amazonas, Mato Grosso, Pará and Rondônia). Some cultivated clones, all of which were derived from the first introduction of rubber trees to Asia, were also included; these clones were grouped together under the category “Wickham” clones. We separately identified genotypes issuing from crosses between some of the Wickham clones and the accessions of wild Amazonian origin.

**Table 1:** Number of accessions per geographical origin and *ex situ* collection site.

	*Ex situ* collection where the accessions were conserved								
Regions from which seeds were collected	Number of sampled locations	CIRAD Guiana	EMBRAPA CPAC	EMBRAPA Manaus	IAC Pindorama	IAC Votuporanga	Michelin Mato Grosso	Unesp Ilha Solteira	Total
Acre	6	93	-	-	21	-	12	61	187
Amazonas	7	-	434	-	-	-	-		434
Madre de Dios	1	8	-	-	-	-	-	-	8
Mato Grosso	5	117	-	-	2	3	11	-	133
Para	2	-	55	-	-	-	-	-	55
Rondônia	8	129	-	-	20	14	23	-	186
Wickham x Amazonian wild	-	29	-	-	-	-	-	-	29
Wickham	-	48	-	-	7	16	-	-	71
Hevea spp.	-	8	-	6	-	-	-	-	14
Total		432	489	6	50	33	46	61	1117

The majority of the accessions studied were assumed to be *H*. *brasiliensis*, with the exception of the following two cases:

- A total of 14 accessions from 7 different species of the genus *Hevea* (4 *H*. *benthamiana*, 2 *H*. *camargoana*, 1 *H*. *guianensis*, 4 *H*. *pauciflora*, 1 *H*. *spruceana*, 1 *H*. *paudari* and 1 *H*. *nitida*) conserved in the *ex situ* collections of Cirad (Guyane) and Embrapa (Manaus). All 14 of these accessions from early surveys have been fully described, and the affiliation of these accessions with the assumed species is not doubted.
- A total of 138 accessions conserved at Embrapa CPAC and collected during the joint Embrapa/RRIM survey of 1995 in Amazonas. These accessions were assumed to originate from *H*. *brasiliensis* × *H*. *guianensis* crosses (100 accessions), *H*. *benthamiana* × *H*. *brasiliensis* crosses (21 accessions), pure *H*. *benthamiana* (13 accessions) or pure *H*. *pauciflora* (4 accessions).

**Fig 1:**
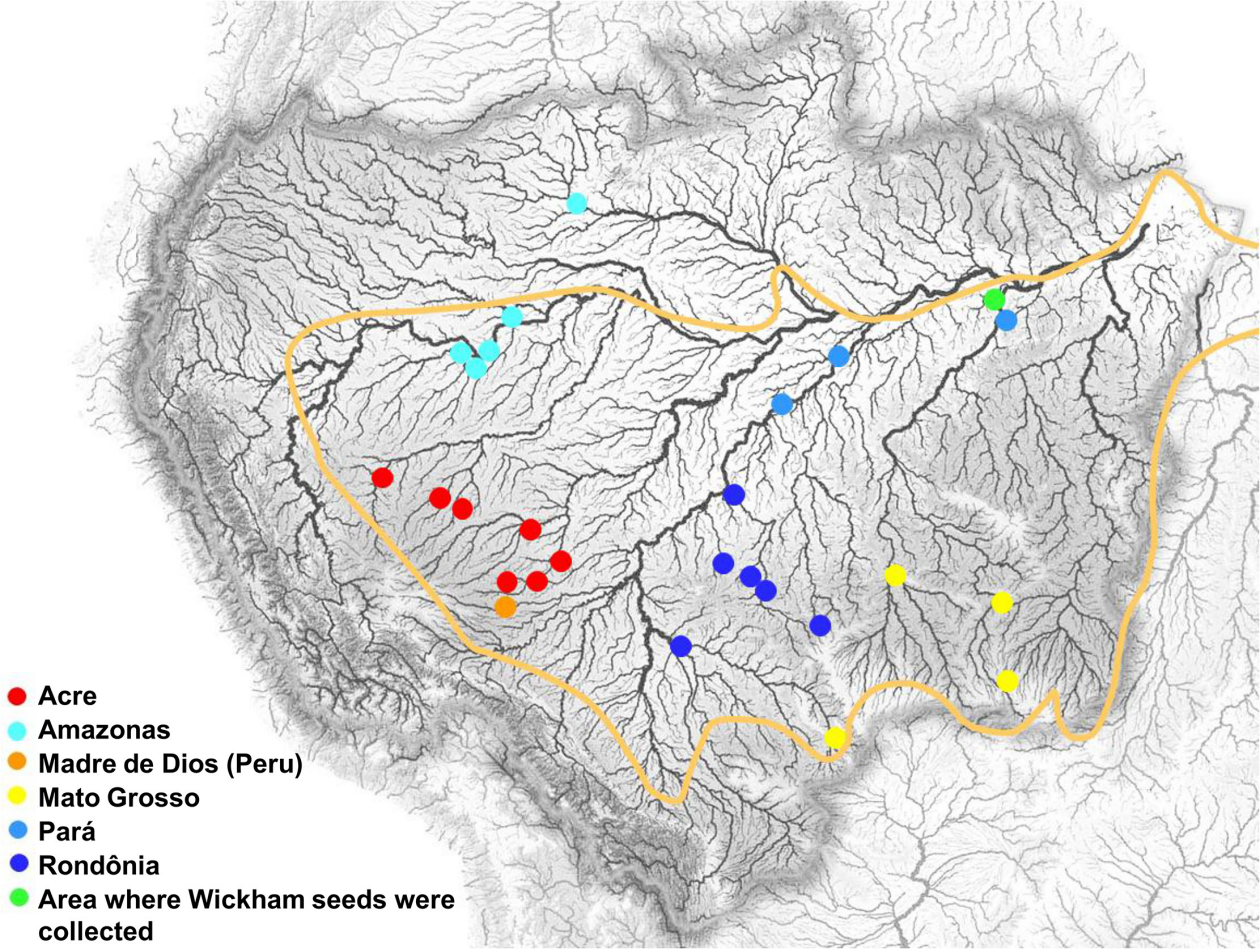
The locations of the sampled sites within Brazil. The colors correspond to the population codes listed in Table 1 and other Figs. This Fig is similar but not identical to the original image from http://www.mappery.com/map-of/Amazon-Basin-Hydrosheds-Map.

For the majority of the accessions studied, passport data indicating the collection site were available. These accessions were collected from a large number of localities within a large geographical sampling area that is representative of the distribution area of the genus *Hevea*. The collection sites are illustrated in Fig 1

The detailed data on the accessions are available in S1 Table. Notably, 52 accessions were putatively analyzed twice (i.e., the accessions appeared under the same name but originated from different collection sites). These repeated accessions will provide further insight into the reliability of the applied methods of genetic analysis and the confidence that can be achieved when conserving the name of an accession across various *ex situ* collections.

### DNA extraction

DNA extraction was performed using two different methods according to the origin of the samples. For accessions from the Cirad (Guyane) collection, DNA was isolated using a standard MATAB process as previously described [23]. For the remaining accessions, the collected leaves were stored at -80°C prior to DNA extraction. Genomic DNA was extracted from leaf disks that were 20 mm in diameter and homogenized in a TissueLyser (Qiagen, Valencia, CA, USA). The DNA was extracted according to the Qiagen DNeasy Plant Mini Kit protocol. The quality and concentration of the extracted DNA were analyzed on 1% (m/v) agarose gels.

### Analysis of molecular markers

A set of 13 microsatellite (simple sequence repeat—SSR) markers (A2365, A2368, A2387, A2389, A2406, A2413, A2508, A2684, A2736, BAC55B02, T2083, TA2163 and TAs2558) developed in previous studies [23] was selected due to the position of the markers in the *Hevea* genetic linkage map, the large number of alleles and the ease of genotyping.

Polymerase chain reaction (PCR) was performed using a three primer labeling system [24] in a final volume of 10 μl containing 25 ng of template DNA, 0.2 μM each primer, 200 μM deoxynucleotide (dNTP), 2 mM MgCl_2_, 1× PCR buffer and 1U of Taq DNA polymerase. The touchdown cycling conditions consisted of an initial denaturing step of 5 min at 94°C, followed by 10 cycles of denaturation, annealing and elongation with a 0.5°C decrease in the annealing per cycle from 55°C to 50°C (94°C for 45 s, 55°C for 1 min, 72°C for 1 min 15 s), 25 cycles with annealing at 50°C (94°C for 45 s, 50°C for 1 min, 72°C for 1 min), and a final elongation step at 72°C for 30 min.

Two different methods were used to separate amplicons based on their migration and visualization. The amplicons from 353 accessions from the CIRAD (Guyane) collection were denatured for 3 min at 94°C, separated on a 6.5% polyacrylamide gel and visualized with a LI-COR 4300 DNA Analyser (LI-COR, Lincoln, NE, USA) as previously described by Le Guen et al. (2009). These 353 accessions included the 307 genotypes analyzed in the above-mentioned study and an additional 46 accessions (mainly from the “*Wickham”* and “*Wickham × Amazonian wild”* categories) that, although they were analyzed using the same method and at the same time, were not mentioned in the previous study. The amplicons from the remaining 764 accessions were denatured with formamide and analyzed with an ABI 3500 (Applied Biosystems, Foster City, CA, USA) Automated Sequencer. The alleles were scored against the internal GeneScan-600 (LIZ) Size Standard Kit (Applied Biosystems, Foster City, CA, USA) using GeneMapper software v.4.1.

### Characterization of genetic diversity

#### Analysis of population genetic parameters

A descriptive statistical analysis for all of the SSR loci and the number of alleles per locus (Na), information index (I), observed heterozygosity (H_O_), expected heterozygosity (H_E_), fixation index (F) and private alleles (Pa) were calculated using GenAlEx v. 6.5 [25]. The confidence intervals of the different parameters were calculated using 1,000 bootstrap iterations.

The analysis of molecular variance (AMOVA) and calculation of F_ST_ and Nei’s G_ST_ among the groups were performed with GenAlEx v. 6.501 [25] to detect the molecular genetic variances within and among the accessions, considering both the collection sites (geographical regions) and the groups suggested by our analysis with the program STRUCTURE. The GenAlEx software was also used to identify private and rare alleles (frequency < 0.05).

#### Genetic structure analysis

Different statistical procedures were used to describe the genetic diversity of the collected germplasm, including (i) a model-based method for inferring population structure by clustering genotypes; (ii) a distance-based model using a dissimilarity matrix; and (iii) a factorial analysis through principal coordinates analysis (PCoA).

The model-based approach consisted of a Bayesian analysis implemented under the STRUCTURE program version 2.3.4 [26,27]. All 1,117 of the genotyped accessions were analyzed using an admixture model with independent allele frequencies in each group. The number of groups (K) to which the genotypes were assigned was systematically tested from 1 to 10; for each value of K, 20 independent iterations of the Bayesian model-based analysis were performed. The burn-in time and replication numbers were set to 100,000 and 1,000,000, respectively, for each run. We used the ΔK *ad hoc* method described by Evanno et al. (2005) and implemented in the online tool Structure Harvester [28] to estimate the most probable K.

After estimating the most probable K, we utilized the greedy algorithm implemented in CLUMPP v.1.1.1 [29]] with a random input order and 1,000 permutations to align the runs. The results were visualized using Distruct v.1.1 [30]. Based on the posterior probability of membership (q) of a given accession belonging to a given group compared with the total (K), we classified individuals showing q > 0.70 as members of a given cluster. In contrast, the accession was classified as admixed in clusters with a membership of q ≤ 0.70. Analyses of secondary structures were performed (with K = 1–10) using only the genotypes corresponding to the clustered group to detect sub-structuring.

The distance-based model was implemented in the DARwin 5.0.158 software [31]. A dissimilarity matrix was computed with a simple-matching index, and a neighbor-joining tree was constructed. A factorial analysis (PCoA) was performed using GenAlEx [25]. These latter methods were used simultaneously to better investigate the precise structure of our sample.

### Core collection sampling

The maximization strategy [32] implemented in the software COREFINDER was used to generate a core rubber tree collection that maximized the number of observed alleles in our dataset. The M-strategy consisted of detecting the sample size that best captured 100% of the genetic diversity present within the entire germplasm collection.

### Ethics statement

We confirm that no specific permits were required to collect the leaves used in this study. This work was a collaborative study developed by researchers from APTA (Brazil), CIRAD (France), EMBRAPA (Brazil), IAC (Brazil), UESB (Brazil) UNESP (Brazil) and UNICAMP (Brazil). Additionally, we confirm that the field studies did not involve endangered or protected species.

## Results

### Data homogenization

As explained in the previous section, the allelic sizes of the PCR products were estimated using the following two technologies: LI-COR technology (electrophoresis in polyacrylamide gels) for 353 accessions and ABI3500 (capillary electrophoresis) for the remaining 764 accessions. Although both technologies are accurate and reliable, deviations may exist between the data obtained from these two sources due to the use of different calibration curves. The analysis of common control genotypes using both technologies allowed us to standardize the sizes of the different allelic products for each microsatellite marker. Moreover, the presence of 52 pairs of accessions among the analyzed samples identified under the same name and analyzed with the two technologies helped us to confirm the goodness of data homogeneity. Among these 52 pairs of homonymic accessions, 26 were identical, 22 exhibited less than 5 differences among the 26 allelic forms (13 microsatellites with 2 alleles each), and 4 differed for at least 5 alleles. Further analyses based on a matrix of dissimilarity (see below) suggested that only these last 4 pairs corresponded to genetically distinct genotypes to which identical names were erroneously assigned.

### Genetic diversity

We computed diversity statistics across all 1,117 of the individual accessions and within each of the two subgroups obtained in the analyses using the STRUCTURE software (Table 1). The overall number of different alleles per SSR locus across all of the accessions ranged from 5 to 39 (data not shown), with a group mean of 14.5 (Table 2).

**Table 2:** Descriptive results of the values observed in the characterization of the mean loci for each population of sample size (N), number of alleles (Na), information index (I), observed heterozygosity (H_o_), expected genetic diversity (H_E_), fixation index (F) and private alleles (Pa) in 1,117 accessions of *Hevea* spp.

Groups^a^	N	Na	I	H_o_	H_E_	F	Pa
Acre (AC)	183	20.0	2.24	0.66	0.81	0.18	11
Amazonas (AM)	428	23.8	2.29	0.65	0.82	0.21	34
Wickham (Wi)	70	9.5	1.39	0.57	0.63	0.09	0
Madre de Dios (MD)	7	6.4	1.53	0.67	0.70	0.05	0
Mato Grosso (MT)	129	14.3	1.88	0.59	0.74	0.23	7
Para (PA)	54	15.8	2.23	0.69	0.84	0.17	3
Rondônia (RO)	183	21.1	2.27	0.63	0.81	0.22	14
Wickham x Amazonian wild (Wi x AM)	28	9.5	1.61	0.64	0.68	0.08	0
*Hevea* spp.	13	10.2	2.05	0.67	0.83	0.18	20
Mean	122	14.5	1.94	0.64	0.76	0.16	10
SE	11.7	0.74	0.06	0.02	0.02	0.01	11.5
Cluster^b^ 1 (red)	24	8.88	2.36	0.67	0.84	0.20	18
Cluster 2 (green)	30	9.04	2.41	0.63	0.84	0.25	97

^a^ The 1,117 accessions were divided according to the locations of origin from which they were collected and assigned to groups.

^b^ Structure analysis results suggest that the set of genotypes can be partitioned into two clusters.

For all of the accessions, the mean observed heterozygosity (H_O_) was 0.64 (ranging from 0.57 to 0.69); this value was slightly higher in cluster 1 than in cluster 2. The genetic diversity (H_E_) was higher than H_O_ in all cases. The analysis revealed a high H_E_ level ranging from 0.63 (Wickham group) to 0.84 (Pará group) with a mean of 0.76. Consequently, the Wright’s fixation index (F) values were positive, with a mean of 0.16 obtained for the accessions overall. The heterozygosities (H_O_ and H_E_) were similar in both of the subgroups, although cluster 2 displayed higher indices of fixation (F = 0.20 in cluster 1 and F = 0.25 in cluster 2).

**Fig 2:**
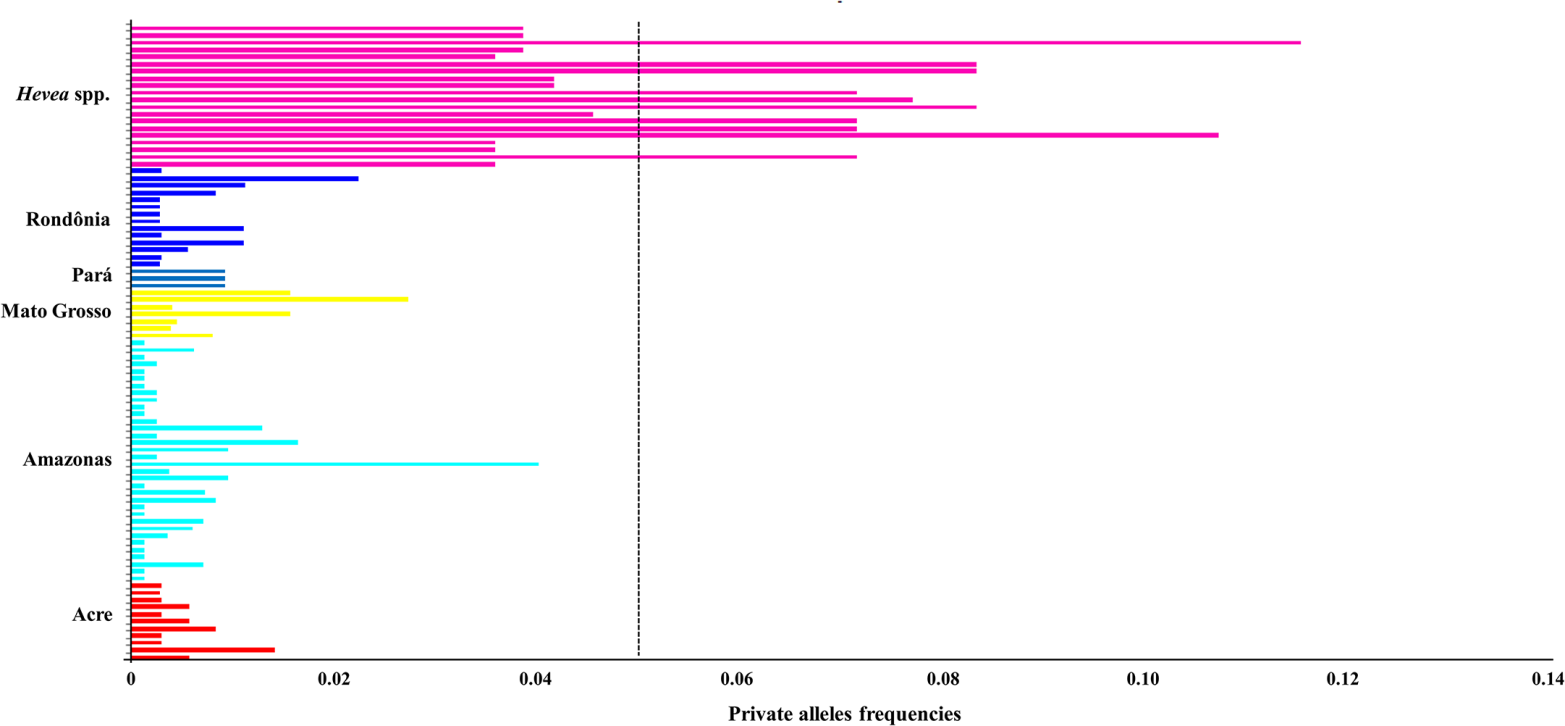
Private allele frequencies obtained from the genotyping of microsatellite loci in the *Hevea* germplasm. The dashed line indicates the cutoff for the occurrence of rare alleles (frequency = 0.05).

Of the 408 observed alleles, 319 alleles were shared among the groups; the remaining 89 alleles represented private alleles in different groups of accessions (Fig 2). The A2508 locus contained the largest number of private alleles of the 13 SSR alleles used in this study (15). The Amazonas population and *Hevea* spp. population exhibited the greatest number of private alleles (34 and 20, respectively). A comparison of the number of private alleles from cluster 1 (18) and cluster 2 (97) showed that the latter contained a larger number of private alleles (Table 2, Fig 2).

### Model-based investigation of genetic structure

**Fig 3:**
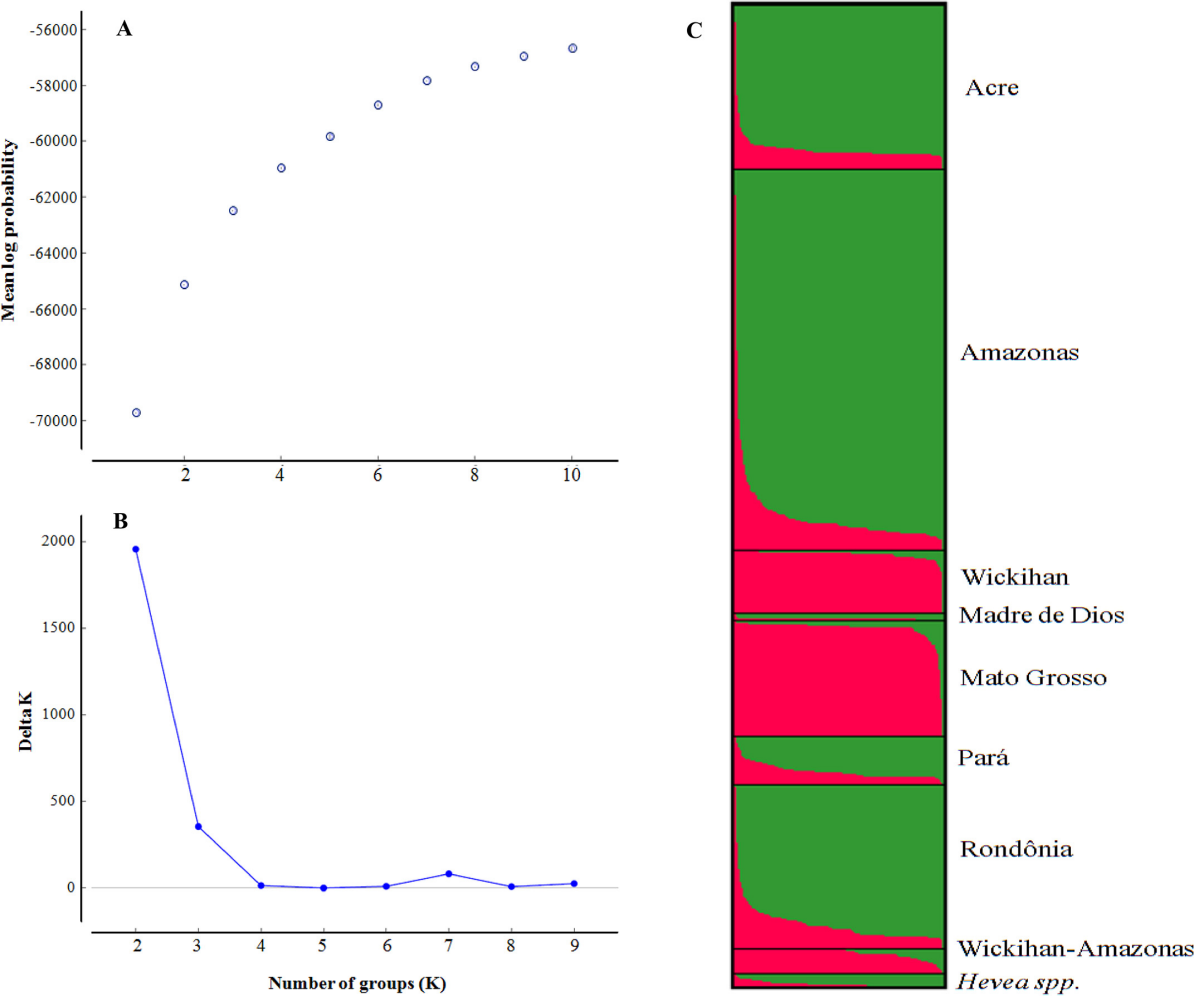
Genetic structure of the *Hevea* germplasm accessions. The genetic structure was inferred through Bayesian analyses of the most probable number of groups (K) estimated based on the mean log probability [A] using the method described by Evanno et al. (2005) [B]. Each column (histogram) [C] represents the genotyping data from one accession, and the colors used in the histogram represent the most likely ancestry of the cluster from which the accessions were derived.

To examine the relatedness among the 1,117 accessions, the data for 13 markers were analyzed using the STRUCTURE program. A strict interpretation of our results using the method of Evanno et al. (2005) suggested that two genetic clusters (K = 2) were sufficient to interpret our data (Fig 3A and 3B); the results are also presented as K = 3 and K = 7 in S3 Fig. Based on a membership probability threshold of 0.70, 277 accessions were assigned to cluster 1 and 783 accessions were assigned to cluster 2. The remaining 57 accessions were classified into a mixed subgroup. The membership probability was <0.70 in any given subgroup (Table 3).

**Table 3:** Division of membership probability in the two clusters (K = 2) inferred based on Bayesian analyses considering the most probable number of groups (K); designated as Cluster 1, Cluster 2 and Mixture (membership q < 0.07).

Groups^a^	Total	Cluster 1 (red)	Cluster 2 (green)	Mixture
Acre (AC)	187	17	165	5
Amazonas (AM)	434	22	400	12
Wickham (Wi)	71	67	2	2
Madre de Dios (MD)	8	1	7	0
Mato Grosso (MT)	133	124	6	3
Para (PA)	55	9	39	7
Rondônia (RO)	186	15	154	17
Wickham x Amazonian wild (Wi x AM)	29	22	0	7
Hevea spp.	14	0	10	4

^a^ The 1,117 accessions were divided according to the locations of origin from which they were collected and assigned to a group.

The level of clustering (K = 2) reflects the primary division of subgroup cluster 1, consisting of the varieties of the advanced breeding germplasm originating from the Wickham and Mato Grosso accessions. Cluster 2 was formed by the wild germplasm from the Acre, Amazonas, Pará and Rondônia populations and *Hevea* spp. A further STRUCTURE analysis of these two primary clusters of genotypes also indicated partitioning of both cluster 1 and cluster 2 into 2 subgroups. (Fig 3). However, no clear pattern for the assignment of individuals based on geographical origin was observed.

**Fig 4:**
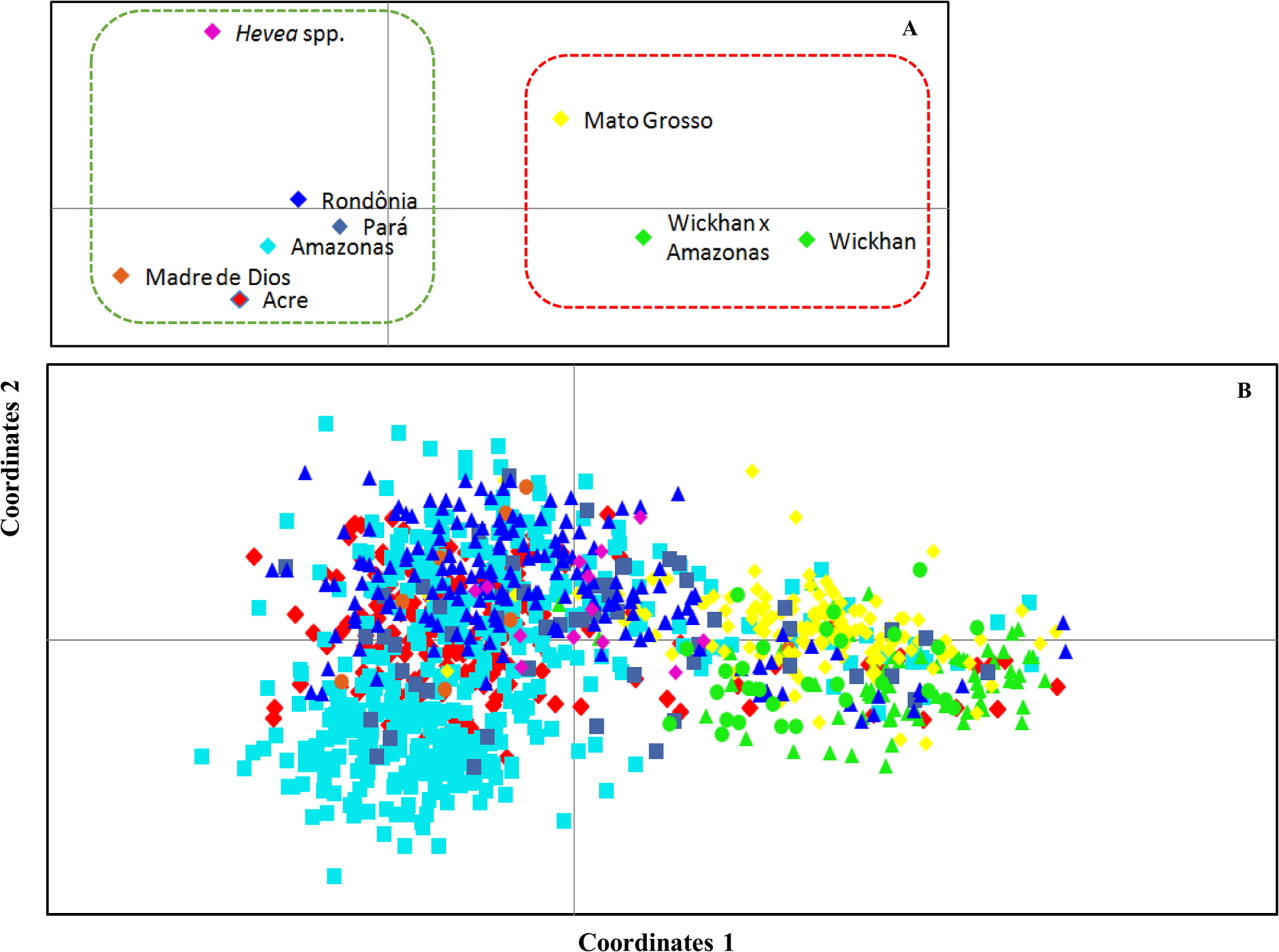
Dispersion graphics based on a principal coordinates analysis (PCoA) of the groups. [A] Accessions (the dashed lines refer to the groups that were observed based on the analysis conducted using STRUCTURE software) [B] from the *Hevea* germplasm based on genotypic data from 13 microsatellite loci.

Additionally, the separation of the groups was also estimated using PCoA (Fig 4A) based on the genetic distance, which divided the entire population into two clear clusters related to the structure analyses. The first and second principal coordinates explained 35% and 20% of the molecular variation, respectively (Fig 4A and 4B). The accessions from Acre, Amazonas, Madre de Dios, Para, Rondônia and *Hevea* spp. were placed into one group, while the remaining populations belonged to the group formed by the Wi, MT and Wi × AM wild accessions.

### Distance-based analysis

The dissimilarity matrix computed with DARwin 5.0.158 was used to identify putative unexpected duplications of genotypes under different accession names. A total of 13 pairs and 2 triplets of accessions were found to be present with a high degree of genetic similarity. All of the concerned accessions were from the oldest and most widespread surveys conducted in 1974 (by EMBRAPA/IRCA) and 1981 (by IRRDB). In the majority of cases, these accessions were registered under almost identical names (e.g., MT/C/5/25 *vs*. MT/C/S/25 or RO/C/9/45 *vs*. RO/C/9/48). Therefore, misidentifications due to human mistakes can be reasonably hypothesized to have occurred during the process of multiplication or transfer of accessions from one *ex situ* collection to another. In two cases, the accession names were completely different.

**Fig 5:**
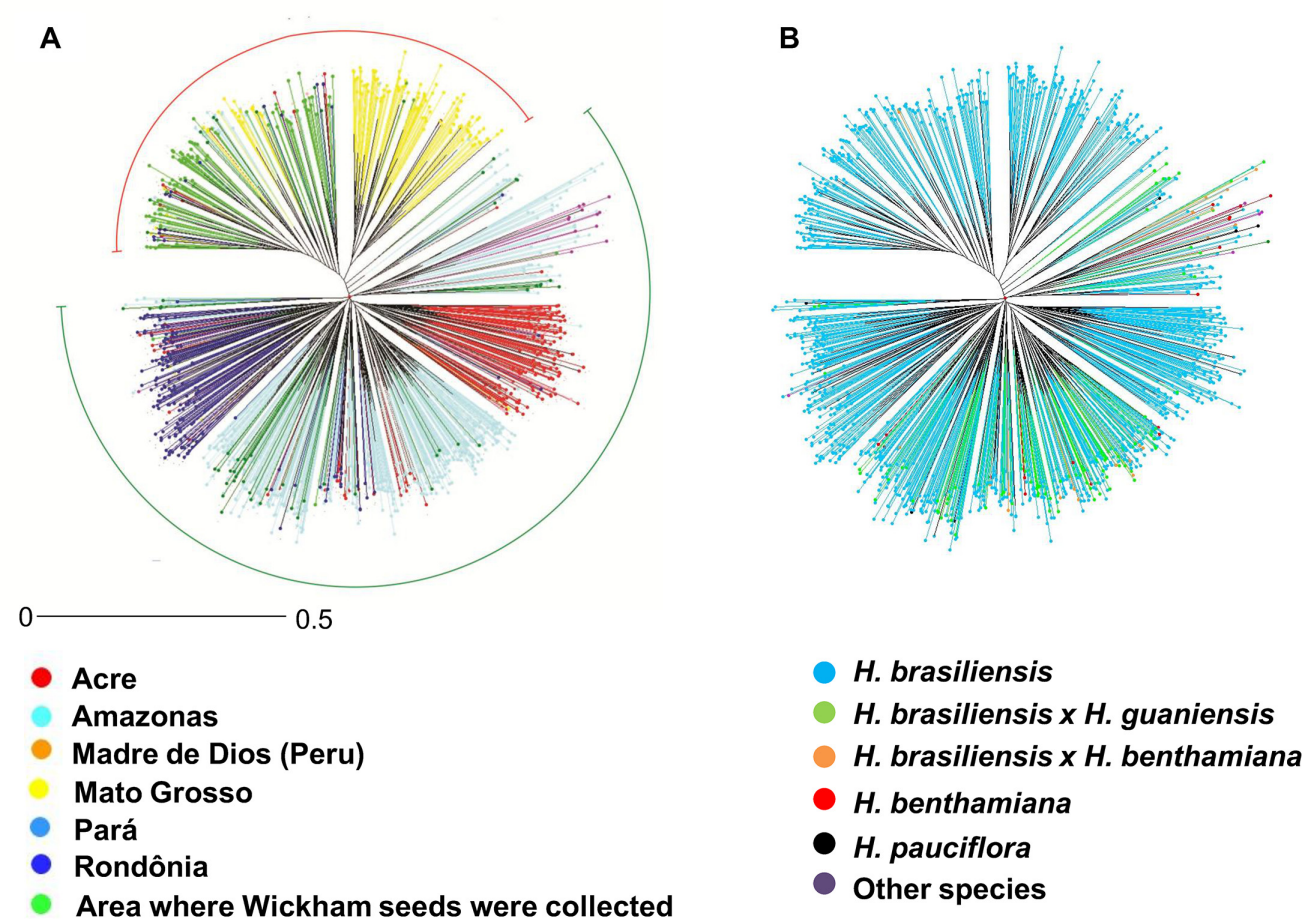
Neighbor-joining tree as a third method of visualizing the relationships between the genotypes. [A]. Classified based on the region of collection. [B]. Classified by the species group.

Finally, we constructed a neighbor-joining tree to confirm the relationships among the accessions. The tree (Fig 5A) showed a pattern that was consistent with the two analyses described above. The group formed by the Wi, MT and Wi × AM wild accessions was clearly separated. The two subgroups suggested by the STRUCTURE analyses were also indicated in the tree. A discrete separation was observed between the genotypes of *H*. *brasiliensis* and other species or between the other species of the genus *Hevea* (13 *H*. *benthamiana* and 4 *H*. *pauciflora*). However, the hybrids (21 *H*. *brasiliensis* × *H*. *benthamiana* and 100 *H*. *brasiliensis* × *H*. *guianensis*) were not separated from the genotypes of the Amazonas group (Fig 5B); these results were confirmed by the PCoA (Fig 4).

We used AMOVA to partition the molecular genetic variance within and among the accessions and between the nine subgroups according to their country of origin. In a preliminary AMOVA, 7% of the total genetic variance was found to occur between the groups, while 21% occurred between the accessions and 73% occurred within the accessions.

Although most of the genetic variance occurred within the accessions, the differentiation of the accessions and groups was highly significant (p ≤ 0.001). The pairwise G_ST_ values (analogous to F_ST_) ranged from 0.008 (between the Amazonas and PA) to 0.099 (between the Wi and MD). Each of the pairwise values was significantly different from zero according to tests based on 9,999 random permutations (p ≤ 0.001) (Table 4). This finding was similar to the F_ST_ values, which ranged from 0.015 (between the AM and PA) to 0.20 (between the Wi and MD). However, the sample sizes from MD are relatively small compared to the other accessions, which may influence the Fst values. In terms of the population structure between the groups recognized in this study, the G_ST_ between cluster 1 and cluster 2 estimated from AMOVA was 0.018 (P < 0.01).

**Table 4:** Triangular matrices for the computation of G_ST_ and F_ST_ tests among the *Hevea* germplasm; G_ST_ values are below the diagonal, and F_ST_ values are above the diagonal.

Groups	AC	AM	Wi	MD	MT	PA	RO	WxAM	*Hevea* sp.
Acre (AC)	—	0.028	0.138	0.036	0.095	0.030	0.044	0.085	0.083
Amazonas (AM)	0.015	—	0.131	0.064	0.089	0.015	0.039	0.088	0.071
Wickham (Wi)	0.081	0.079	—	0.201	0.086	0.100	0.134	0.036	0.179
Madre de Dios (MD)	0.010	0.025	0.099	—	0.132	0.061	0.076	0.117	0.086
Mato Grosso (MT)	0.053	0.049	0.047	0.063	—	0.061	0.076	0.056	0.109
Pará (PA)	0.016	0.008	0.054	0.027	0.032	—	0.033	0.058	0.065
Rondônia (RO)	0.023	0.020	0.078	0.031	0.041	0.017	—	0.089	0.076
Wickham x Amazonian wild (WIxAM)	0.049	0.051	0.018	0.057	0.031	0.032	0.050	—	0.124
*Hevea* sp.	0.043	0.036	0.091	0.052	0.055	0.034	0.038	0.067	—

### Rubber tree core collection

**Fig 6:**
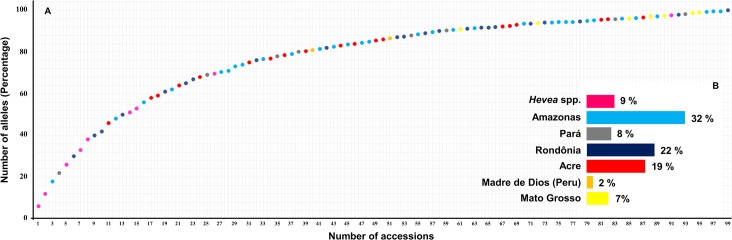
Genetic diversity (expressed as a percentage of the total number of alleles) as a function of the number of accessions included in the rubber tree germplasm core collection.

A core collection was assembled for the rubber tree germplasm to represent all of the genetic diversity identified in this study. The 408 alleles identified at the 13 microsatellites used in this study were fully represented by as few as 99 accessions (the best of 10 runs) (Fig 6 and S1 Table). The core collection was represented by the accessions from Amazonas (32.3%), Rondônia (22.2%), Acre (19.2%), *Hevea* spp. (9.1%), Para (8.1%), Mato Grosso (7.1%) and Madre de Dios (2%). The alleles identified in this study were fully represented in these core collections. Additionally, our COREFINDER analysis highlighted that 85% (84 accessions) of the entire core collection was represented by the rubber tree accessions grouped in cluster 2, whereas cluster 1 contributed a smaller percentage (11%) to the core collection; the accessions that were considered a mixture (classified into this subgroup by Structure analysis) contributed to only 4% (4 accessions) of the core collection.

## Discussion

### The history of the collections

The present study is the most comprehensive study ever performed on the population genetics of the genus *Hevea*. This study was based on more than 1,100 accessions sampled at 7 *ex situ* collection sites located in Brazil and French Guiana. Nearly 500 accessions from the collecting survey conducted in 1995 in the Brazilian states of Amazonas and Pará that had never been previously described were included in this study. The other accessions mostly came from the states of Acre, Mato Grosso or Rondônia and were obtained in surveys performed in 1974 and 1981. Since that time, the accessions have been conserved in different *ex situ* collections. In recent decades, this latter group of accessions has potentially been transferred from one collection site to another, with all of the possible associated risks regarding traceability. For example, the accessions kept at the French Guiana collection site were primarily contributed by the CNRA collection site at Bimbresso in Côte d’Ivoire, where they had been sent soon after the respective prospection [20]. Some of these accessions were subsequently distributed from French Guiana to the Michelin collection in Brazil. Therefore, the first use of highly polymorphic microsatellite markers to characterize this material was to determine the overall consistency of the accession denominations. The comparison of 52 pairs of homonymic accessions conserved at different sites allowed us to conclude that only 4 corresponded to distinct genotypes. Moreover, 13 pairs (and 2 triplets) of accessions with differing identifications presented highly similar genotypes, suggesting loss or modification of their true identity across the transfer of accessions between collection sites. Notably, these anomalies in identification were detected only for old accessions that were transferred from site to site through grafted plants. The more recent accessions (i.e., from Amazonas and Pará in EMBRAPA/CPAC or from Acre in UNESP Ilha Solteira) that have not yet been multiplied did not present these anomalies. Therefore, microsatellite markers appear to be useful tools for the management of accessions in collections, especially when transfers or exchanges of biological material between sites are scheduled.

### Genetic diversity

The results from this study reveal high levels of genetic diversity among the accessions, which is consistent with previous studies [23,33] and the biology of this outbred plant species. We detected an average of 14.5 alleles per locus and an H_o_ of 0.64 across the entire accession set. These values were slightly higher than those detected by Le Guen et al. (2009) (H_o_ = 0.59). Lam et al. (2009) have detected an average heterozygosity of 0.20 using random amplified polymorphic DNA (RAPD) markers in *Hevea* genetic resources in Vietnam. These differences in genetic diversity values might be attributable to the types of germplasm that were analyzed. Lekawipat et al. (2003) have used microsatellite markers to detect the diversity in 40 Wickham and 68 Amazonian accessions using 170 alleles from 12 microsatellite markers dispersed among all of the genotypes. Similar results were observed in our study, in which an average of 14 alleles were available per locus; this diversity can be attributed to the high variability of Amazonian accessions.

The estimates of G_ST_ and F_ST_ values revealed low genetic differentiation between the groups, with the exception of the Wickham accessions, which showed greater differentiation compared to the other studied groups (Table 4). The Wickham accessions originated from breeding programs with a narrow genetic base [8,34]; thus, the Wickham accessions were unambiguously less variable than the Amazonian accessions, thus causing the inflation of Fst.

An outcrossing breeding system, long life cycle and large geographic range may have helped to shape the high genetic diversity of *Hevea* plants. Le Guen et al. (2009) have obtained a similar value (F_ST_ = 0.12) upon analyzing *ex situ* collections of wild rubber trees. Gouvea et al. (2010) have studied clones from controlled-cross and open pollinated progenies and reported high values of genetic differentiation (G_ST_ = 0.61). Lower genetic differentiation indicates higher gene flow between populations, indicating that a majority of the genetic diversity is preserved within populations.

A population structure analysis using the software package STRUCTURE revealed the presence of two primary groups in our set of accessions. This result partially agrees with the results obtained through PCoA (Fig 4) analysis, in which the genotypes from *Hevea spp*. were discretely separated from the other populations to form cluster 2. However, due to the small number of samples analyzed (only seven *Hevea* spp. genotypes were included in this group), this result should be interpreted with caution. The observed heterozygosity was slightly higher in cluster 1 (Wi, Wi × AM and MT accessions) than in cluster 2 (formed by the other analyzed accessions); nevertheless, the number of different alleles was greater in cluster 2, which can be explained by the greater diversity of the different species present in this cluster.

Prior analyses by Le Guen et al. (2009) have demonstrated a distinct separation between the Acre and Rondônia groups and the Mato Grosso group. Although only a few Wickham accessions were included among the samples, these clones appeared to be closer to the Mato Grosso accessions. Our analysis of 55 additional Asian accessions confirmed the genetic similarity between the Wickham and Mato Grosso accessions. The most obvious division in the analyzed accessions occurred between the Mato Grosso and Wickham clones; these clones formed one group, while the other accessions grouped together in a second cluster.

One explanation for this result is that the accessions are from geographically distant populations. However, these two groups are located in the same hydrographical network, which originates downstream of the Tapajos River [23,34,35].

Determining a structure within the accessions of Amazonas, Rondônia, Acre, Madre de Dios and Pará is more difficult. One possible explanation for this difficulty is that long-distance gene flow via seed flotation prevented the isolation of Amazonian populations of *H*. *brasiliensis* separated by distances within catchments and incurred the isolation in populations with distances that exceeded the size of a catchment [23]. The rubber tree is pollinated by midges and thrips, and *He*v*ea* pollen can generally be dispersed by these pollinating agents in the course of open pollination. Yeang et al. (1999) [36] have estimated that the proportion of crosses and selfs changes with distance, and *He*v*ea* pollen can be transported fairly considerable distances by pollinating agents. The regression model in this study suggested a theoretical range of 0±3 to 1±1 km, although very few pollen grains would be expected to reach this limit.

The first study aiming to understand the genetic diversity of the wild *Hevea* germplasm was conducted using isozyme markers in accessions from the 1981 IRRDB collection and Wickham group. The results of the isozyme analysis detected a total of 60 alleles in the 1981 IRRDB accessions and 26 alleles in the Wickham accessions. Based on these results, Chevallier (1988) affirmed that the genetic base of the *Hevea* germplasm 1981 IRRDB collection was prosperous and diversified. In contrast, the Wickham population showed a low level of genetic variability, which is a consequence of oriented selection within a narrow geographical origin over a period of years. Our results demonstrated a greater number of private alleles in cluster 2, which is composed of the wild germplasm (Table 2, Fig 2), thus confirming that the wild germplasm accessions bear most of the rubber tree genetic diversity present in the collections.

Other studies have subsequently been performed on the same 1981 IRRDB population using restriction fragment length polymorphism (RFLP) markers [35,37] and PCR-derived markers [23,34]. This collection can contribute effectively to the long-term progress of the *Hevea* breeding and selection program due to its high genetic variability.

In 1995, an expedition was launched by RRIM to collect rubber seeds from Brazil. From this collection, approximately 50,231 seedlings were planted in Malaysia, including other species of the *Hevea* genus. The first study using this material selected 150 genotypes to identify the most promising rubberwood-yielding genotypes and wild *Hevea* relatives conserved in the 1995 *Hevea* germplasm in Selangor, Malaysia. The genotypes and wild relatives that are considered vigorous and generate a high rubberwood yield can be exploited as promising planting material in breeding programs. In short, the expedition to collect wild *Hevea* relatives in 1995 was a worthwhile scientific activity to safeguard the most valuable genetic resources and improve future crops of the *Hevea* species [22].

Although this *ex situ* germplasm collection presents great genetic potential for breeding, the collection remains underutilized. AMOVA demonstrated that 3% of the total genetic variance was present between the groups and 97% was within the groups, which could be explained by the large number of analyzed individuals in each group. Although the majority of the genetic variation occurred between the individuals within a group, the differences between the accessions and group differentiation were significant (p < 0.001). Our analysis revealed a high frequency of gene flow between the groups, with a genetic differentiation coefficient (G_ST_) estimated to be 0.018.

The majority of the germplasm collection used in the current breeding programs was derived from the 1981 IRRDB collection expedition in the Amazon forests of Brazil, but 434 clones from the 1995 germplasm collection (Amazonas genotypes) showed 34 private alleles and high genetic variability, which stands out among the groups. Altogether, the groups contained 55 private alleles, of which 20 alleles were from the *Hevea* spp. group. These genotypes may be incorporated in future breeding programs.

### A species complex

In addition to the high gene flow detected, no distinct separation among the *H*. *brasiliensis* accessions and the other species from Amazonas was observed in the Structure analysis. However, using PCoA analysis, a possible discrete separation of *Hevea* spp. genotypes from the other genotypes of cluster 2 was observed. The results obtained by Lam et al. (2009) suggest that gene flow might exist between the districts, possibly due to the species’ outcrossing mode of reproduction and the dispersion of seeds by a network of rivers in the Amazon basin. However, this explanation should be treated with caution due to the small number of samples analyzed in that study.

In the present work, several Amazonas accessions were originally identified as other *Hevea* species (13 *H*. *benthamania* and 4 *H*. *pauciflora*) or interspecific hybrids (21 *H*. *benthamiana × H*. *brasiliensis* and 100 *H*. *brasiliensis × H*. *guianensis*). This previous identification was based on the seed size, shape and layout pattern of the accessions. However, whether the trees from which the seeds were collected were hybrids or pure species pollinated by other species is not clear. No methods exist to easily determine whether an *H*. *brasiliensis* seed was generated by pollination from *H*. *brasiliensis* or *H*. *guianensis*. Our difficulty in structuring the Amazonas populations, even within the *H*. *brasiliensis* genotypes, mostly likely occurs because we are not observing a single species but a species complex consisting of at least *H*. *brasiliensis*, *H*. *guianensis* and *H*. *benthamiana*.

Since the publication of the first papers on the subject, the *Hevea* species have been generally assumed to be freely interfertile [38,39]. Pires (1981) has observed natural hybrids of *H*. *camargoana* × *H*. *brasiliensis*, and Gonçalves et al. (1982) have analyzed the progenies that issued from hand pollination through this type of crossing. Additionally, *Hevea* species might be considered a species complex due to the absence of a strict barrier to recombination between the species.

Recently, studies regarding the cross-species/genera transferability of SSR markers have been reported [40–43]. According to Saha et al. (2005), the cross-fertility potential and high SSR transferability observed in *Hevea* species indicate that the genus is a species complex with moderate differentiation between species. Moreover, the results suggest that these aspects are favorable for genetic introgressions using other *Hevea* species in the rubber breeding population, which is mainly based on *H*. *brasiliensis*.

### A core collection to capture allelic diversity

The conventional solution for the conservation of *Hevea* genetic resources has been the establishment of *ex situ* gene banks that can represent a last resort for many species and varieties (including those of the *Hevea* genus), which would otherwise die out as their habitats are destroyed [33]. The conversion of tropical rain forests to pasture and cropland is having dramatic effects on the environment. Over the past several years, the native landscape in the Amazon basin has been rapidly and radically transformed by human actions. Particularly intense and rapid deforestation is taking place in the state of Rondônia, Brazil, that can be observed in a series of satellite images (S1 and S2 Figs).

In this work, 186 *Hevea spp*. accessions were collected from 8 Rôndonia regions that have most likely been deforested during the last 30 years, resulting in a huge loss of the important *Hevea spp*. germplasm. Considering the deforestation scenario in Amazonia (and particularly in Rondônia), the establishment and *ex situ* conservation of *Hevea spp*. germplasm collections is the last possibility to preserve their genetic diversity.

The purpose of core collections is to facilitate the use, maintenance and management of the germplasm by providing a set of accessions that display most of the genetic diversity available in the larger collection with minimum redundancy [44]. Such strategies have already been developed for important perennial crops such as apple trees [45], olive trees [2] and fig trees [46]; these collections are essential for the rational utilization of the germplasm scattered among numerous *ex situ* collection sites in different countries.

Various *Hevea* gene banks exist worldwide, mostly on the South American continent. However, the majority of these collections are composed of accessions from the 1981 IRRDB survey [47]. None of these accessions contain as much diversity as described in the present study for the accessions collected during the last extensive survey performed in 1995 in the Brazilian states of Pará and Amazonas. The present study addressed this topic by providing a molecular database that should facilitate the management of the *Hevea* germplasm. Moreover, we identified a large number of private alleles and high genetic variability in the accessions from the 1995 germplasm. Although the planted and recently domesticated populations exhibited greater genetic diversity, the wild populations have preserved the most private and rare alleles, causing these populations to be the most important reservoirs of genetic variation.

The selection procedure for developing a *Hevea* core collection resulted in the selection of 99 accessions from the germplasm collection with 85% of the entire core collection represented by the rubber tree accessions grouped in cluster 2. The observed heterozygosity was slightly lower in cluster 2 than in cluster 1, but the number of private alleles was greater in cluster 2, as predicted. These observations can be explained by the greater diversity of the different species present in cluster 2 and the greater contribution to the core collection. A total of 32.3% of the accessions were from the Amazonas group, indicating the importance of the wild *Hevea* relatives collected in 1995 and confirming that these collection efforts were a worthwhile scientific activity to safeguard the most valuable genetic resources for future crop improvements of the *Hevea* species.

The accessions of the proposed core collection comprise 85% wild material, and the collection would possess abundant genetic diversity and private alleles, an advantageous attribute for dissecting the genetic basis of QTL for immediate application in *Hevea* breeding.

These investigations should include complementary criteria, such as phenotypic, agronomic and adaptive traits. A core collection would also provide a logical subset of germplasm for examination when the entire collection cannot be used.

Finally, such core collections would also be useful for the development of new breeding strategies for adaptive and agronomic traits through genome-wide association studies.

## Supporting Information

S1 Fig Satellite images of the state of Rondônia in western Brazil. (A) June, 1975; (B) July, 1986; (C) August, 2001; (D) August, 2013; red dot–city of Ariquemes. As much as 67,764 km^2^ of rain forest had been cleared through 2003. Systematic cutting of forest vegetation starts along the roads and then fans out to create the "fishbone" pattern that begins to be visible in the eastern half of the 1986 image. The deforested land and urban areas appear lavender; healthy vegetation appears green. (http://earthshots.usgs.gov/earthshots/node/39#ad-image-0). Images courtesy of the Geological Survey (the USGS home page is: http://www.usgs.gov.)
(TIFF)

S2 Fig Deforestation branching off from roads in the city of Ariquemes, state of Rondônia, in western Brazil. (A) June, 1975; (B) July 1986; (C) August, 2001; (D) August, 2013. The early images show main roads cutting through the forest. Highway 421 snakes through the forest south-southwest from the city of Ariquemes, and Highway 364 runs roughly north to south through Ariquemes. Additional roads branch out from the main roads to create the fishbone pattern. As time proceeds, a patchwork of cleared areas, forest remnants, and settlements are left behind. (http://earthshots.usgs.gov/earthshots/node/39#ad-image-5). Images courtesy of the Geological Survey (the USGS home page is: http://www.usgs.gov.
(TIFF)

S3 Fig Bayesian clustering results. Bar plots from the CLUMPP results aligning 20 structure runs for K = 3 and K = 7.
(TIFF)

S1 Table Population sampled in this study with the state origin, putative geographic origin, number of accessions sampled and genotyping results obtained with the 13 microsatellite markers. (XLSX)
