# Supplementary figures and images for: Genetic Diversity Strategy for the Management and Use of Rubber Genetic Resources: More than 1,000 Wild and Cultivated Accessions in a 100-Genotype Core Collection
Source: PLoS One. 2015 Jul 30;10(7):e0134607. doi: 10.1371/journal.pone.0134607 (PMC4520663; doi:10.1371/journal.pone.0134607)

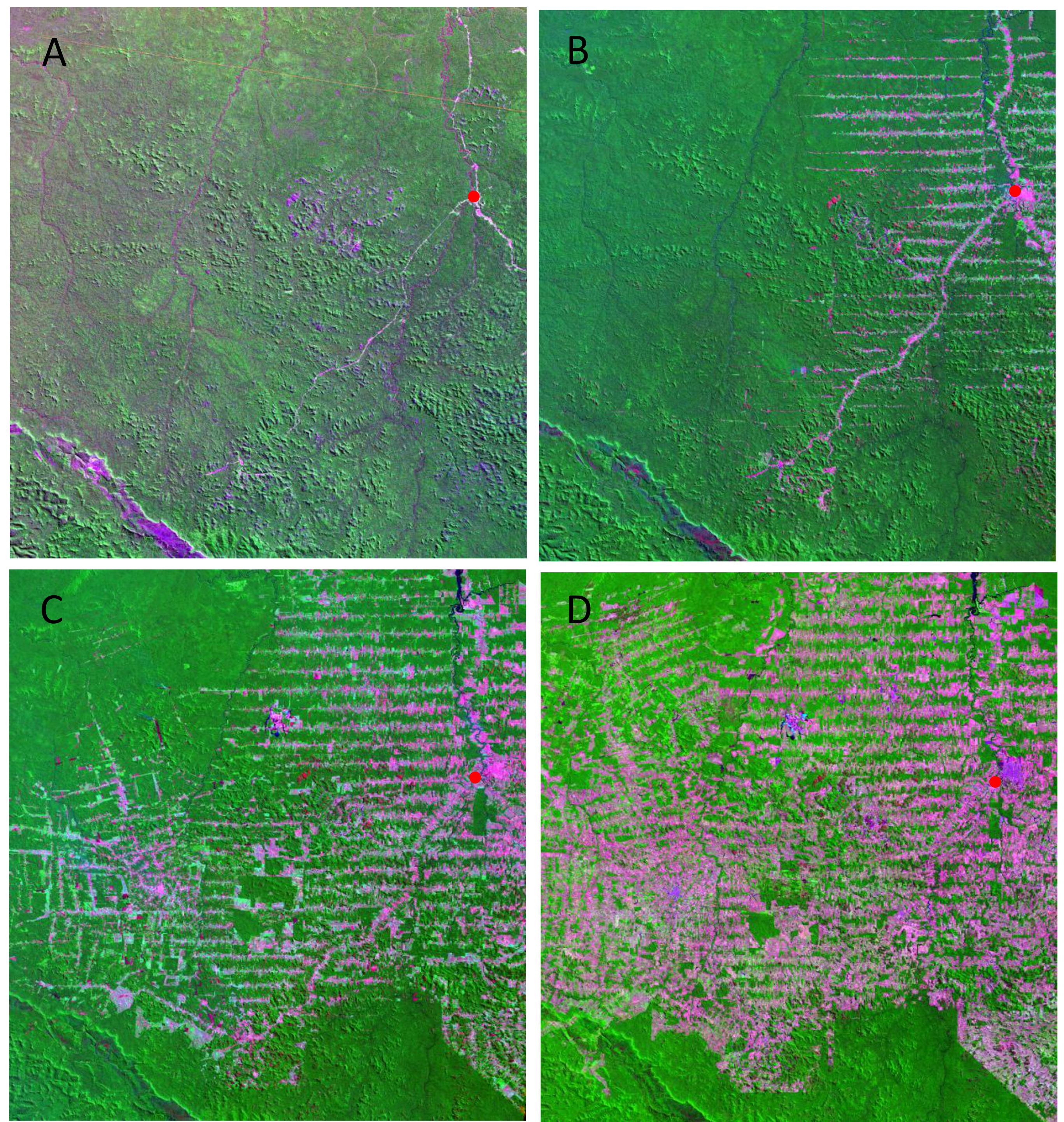

Supplement: S1 Fig — (A) June, 1975; (B) July, 1986; (C) August, 2001; (D) August, 2013; red dot–city of Ariquemes. As much as 67,764 km2 of rain forest had been cleared through 2003. Systematic cutting of forest vegetation starts along the roads and then fans out to create the "fishbone" pattern that begins to be visible in the eastern half of the 1986 image. The deforested land and urban areas appear lavender; healthy vegetation appears green. (http://earthshots.usgs.gov/earthshots/node/39#ad-image-0). Images courtesy of the Geological Survey (the USGS home page is: http://www.usgs.gov.) (TIFF) [file pone.0134607.s001.TIFF]

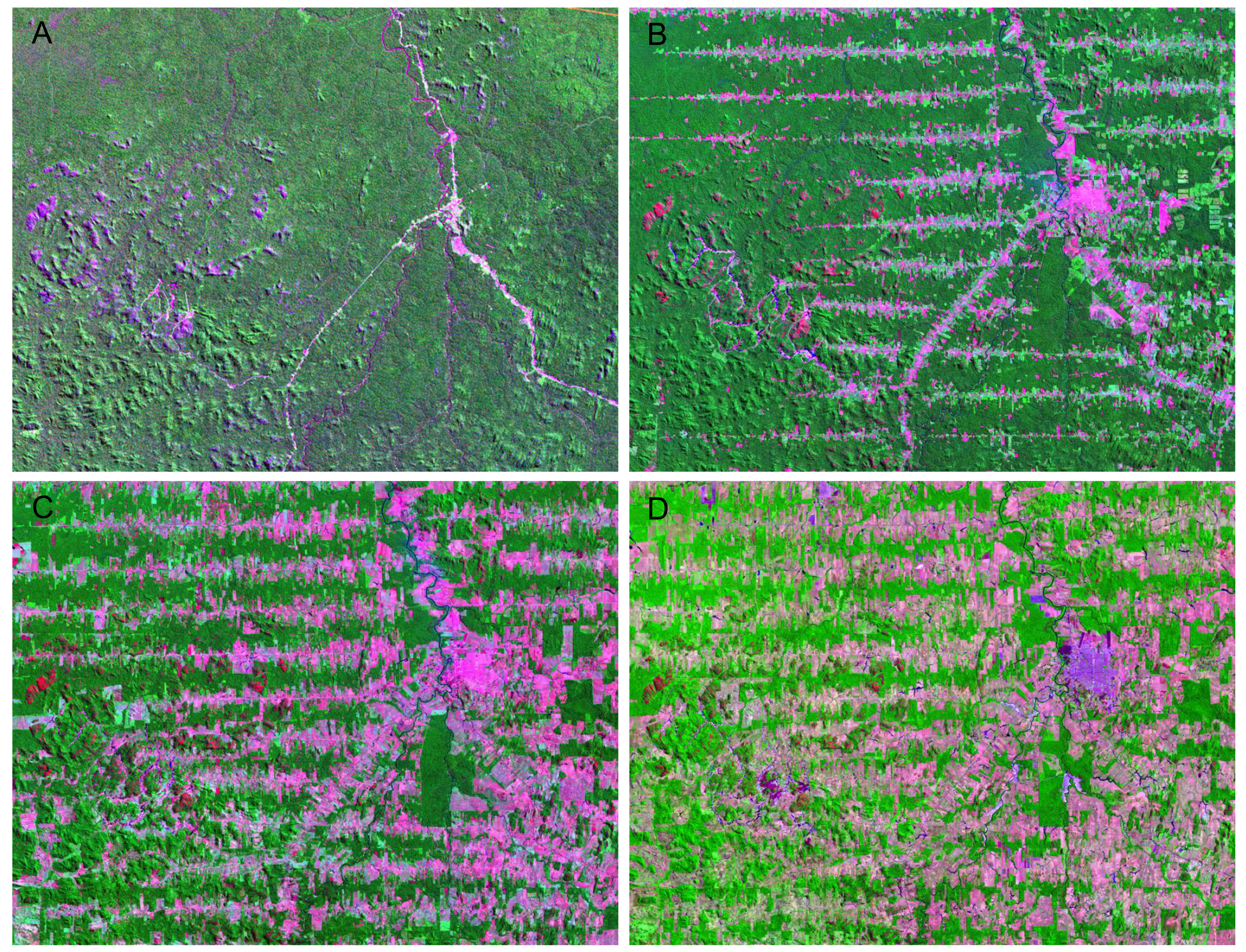

Supplement: S2 Fig — (A) June, 1975; (B) July 1986; (C) August, 2001; (D) August, 2013. The early images show main roads cutting through the forest. Highway 421 snakes through the forest south-southwest from the city of Ariquemes, and Highway 364 runs roughly north to south through Ariquemes. Additional roads branch out from the main roads to create the fishbone pattern. As time proceeds, a patchwork of cleared areas, forest remnants, and settlements are left behind. (http://earthshots.usgs.gov/earthshots/node/39#ad-image-5). Images courtesy of the Geological Survey (the USGS home page is: http://www.usgs.gov. (TIFF) [file pone.0134607.s002.tiff]

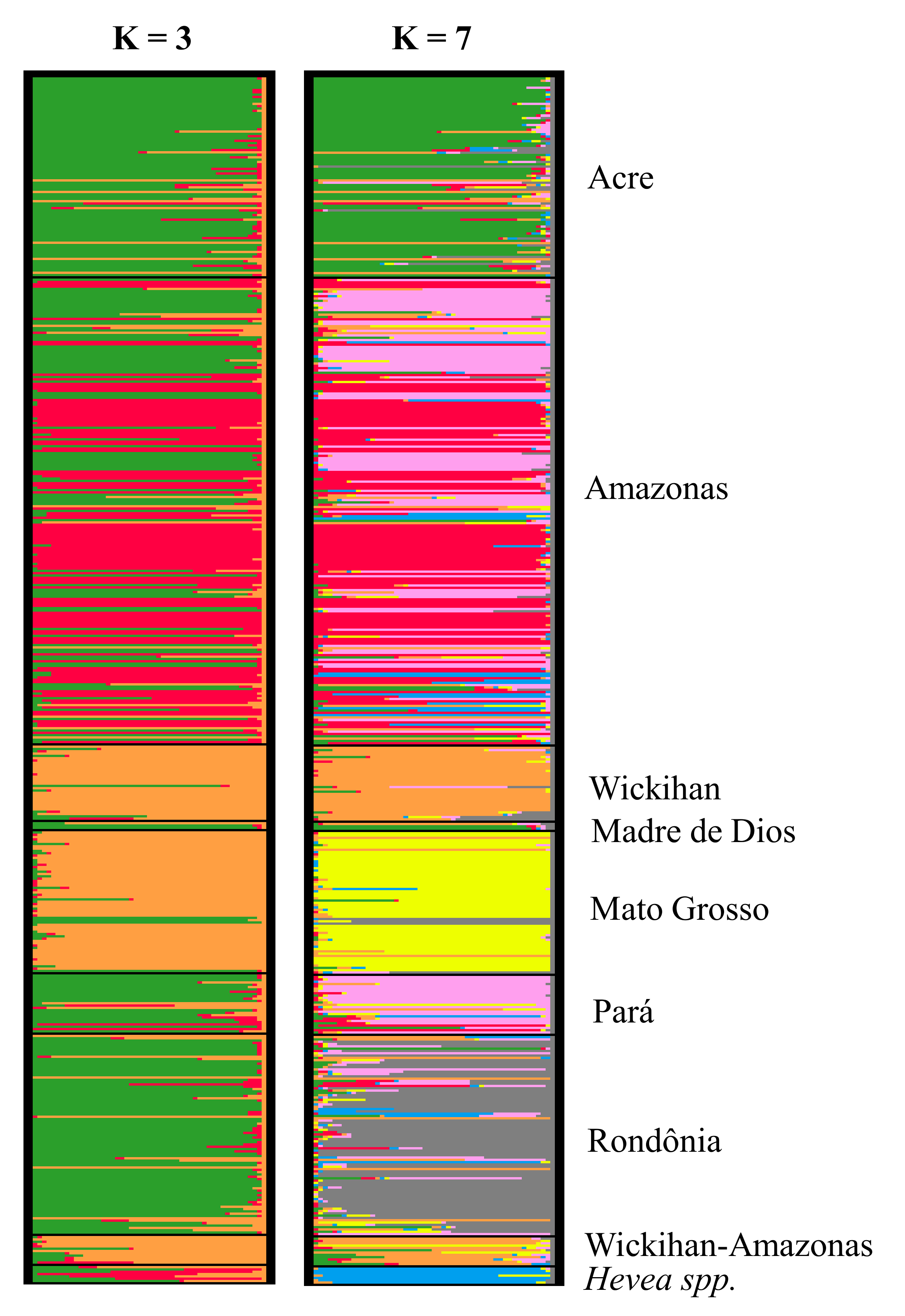

Supplement: S3 Fig — Bar plots from the CLUMPP results aligning 20 structure runs for K = 3 and K = 7. (TIFF) [file pone.0134607.s003.tiff]
